# Seaweed-Based Molecules and Their Potential Biological Activities: An Eco-Sustainable Cosmetics

**DOI:** 10.3390/molecules26175313

**Published:** 2021-09-01

**Authors:** Haresh S. Kalasariya, Virendra Kumar Yadav, Krishna Kumar Yadav, Vineet Tirth, Ali Algahtani, Saiful Islam, Neha Gupta, Byong-Hun Jeon

**Affiliations:** 1 Microbiology Department, Sankalchand Patel University, Visnagar 384315, Gujarat, India; 2 Department of Engineering, River Engineering Pvt. Ltd., Ecotech Phase III, Greater Noida 110042, Uttar Pradesh, India; 3 Faculty of Science and Technology, Madhyanchal Professional University, Ratibad, Bhopal 462044, Madhya Pradesh, India; envirokrishna@gmail.com; 4 Mechanical Engineering Department, College of Engineering, King Khalid University, Abha 61411, Asir, Saudi Arabia; vtirth@kku.edu.sa (V.T.); alialgahtani@kku.edu.sa (A.A.); 5 Research Center for Advanced Materials Science (RCAMS), King Khalid University Guraiger, Abha 61413, Asir, Saudi Arabia; 6 Civil Engineering Department, College of Engineering, King Khalid University, Abha 61413, Asir, Saudi Arabia; sfakrul@kku.edu.sa; 7 Institute of Environment and Development Studies, Bundelkhand University, Jhansi 284128, Uttar Pradesh, India; nhgupta83@gmail.com; 8 Department of Earth Resources and Environmental Engineering, Hanyang University, Seoul 04763, Korea

**Keywords:** cosmeceuticals, seaweeds, skin cosmetics, marine macroalgae, biological activities

## Abstract

Amongst the countless marine organisms, seaweeds are considered as one of the richest sources of biologically active ingredients having powerful biological activities. Seaweeds or marine macroalgae are macroscopic multicellular eukaryotic photosynthetic organisms and have the potential to produce a large number of valuable compounds, such as proteins, carbohydrates, fatty acids, amino acids, phenolic compounds, pigments, etc. Since it is a prominent source of bioactive constituents, it finds diversified industrial applications viz food and dairy, pharmaceuticals, medicinal, cosmeceutical, nutraceutical, etc. Moreover, seaweed-based cosmetic products are risen up in their demands by the consumers, as they see them as a promising alternative to synthetic cosmetics. Normally it contains purified biologically active compounds or extracts with several compounds. Several seaweed ingredients that are useful in cosmeceuticals are known to be effective alternatives with significant benefits. Many seaweeds’ species demonstrated skin beneficial activities, such as antioxidant, anti-melanogenesis, antiaging, photoprotection, anti-wrinkle, moisturizer, antioxidant, anti-inflammatory, anticancer and antioxidant properties, as well as certain antimicrobial activities, such as antibacterial, antifungal and antiviral activities. This review presents applications of bioactive molecules derived from marine algae as a potential substitute for its current applications in the cosmetic industry. The biological activities of carbohydrates, proteins, phenolic compounds and pigments are discussed as safe sources of ingredients for the consumer and cosmetic industry.

## 1. Introduction

Cosmeceuticals are defined by cosmetic producers as products to improve or alter the skin functions and appearance, causing skin benefits [1]. The term “cosmeceutical” refers to products that can combine both cosmetic and pharmaceutical uses to improve skin characteristics, such as the appearance, structure, and functions of the skin [2,3]. This cosmeceutical sector is highly innovative and always looking for principally active molecules that serve better characteristics to open up many possibilities [4]. The cosmetic sector continues to develop in many developing countries that started by the global beauty market [5]. Based on this encouraging future, the production of cosmetics formulations without any side effects is practiced to satisfy the customers [6,7]. The ever-expanding market of the cosmetic industry has led to the use of synthetic or chemical compounds for economic benefits [8]. These harmful components interact with the skin layers and produce toxicological effects on the living body [9,10]. There are many synthetic chemicals occupying space in cosmetic preparation, such as BHA (Butylated hydroxyanisole), BHT (Butylated hydroxytoluene), Coal tar dyes, diethanolamine (DEA), dibutyl phthalate(DBP), parabens, perfume, polyethylene glycol (PEG), petrolatum, siloxane, and heavy metals [11,12,13,14,15,16,17,18,19]. These components accumulate in the skin layers and cause many dermatological conditions, such as dermatitis, cancers, skin rashes, multiple stretch marks, yellowish-brown coloration, etc. [20,21]. Because of these harmful side effects, the use of cosmetics products has become a serious public health problem. This type of use with prolonged exposure on skin accumulates and causes harmful effects, such as an allergic reaction, irritation, and exfoliation [22]. Hence, the goal is to reduce and remove these undesired effects by using natural resources as an alternative in cosmetic formulations to meet consumer demands. Natural resources offer many more advantages, as they are environmentally friendly, are less toxic, are non-carcinogenic, are easily accessible, have lesser side effects, and are economically beneficial [23,24,25]. These include terrestrial plants, animals, and heterogeneous groups in the oceans, etc., with their richness as a source of biologically active principles [26]. A variety of marine organisms, such as fishes, seabirds, reptiles, marine mammals, seaweeds, and many other sources, have been examined by researchers to identify and extract various biologically active constituents, and they have found that all resources are an exceptional reservoir for potential ingredients [27,28]. Among diversified marine organisms, seaweeds are utilized as one of the most significant sources with wide potentials, alternatively [29]. Seaweeds are a novel source of potentially active compounds (proteins–lectins, phycobiliproteins, peptides, amino acids, polyphenols, and polysaccharides) to be exploited in human health benefits, such as antiviral, anticancer, anticoagulant, anti-obesity, and diabetes modulator [30]. Shannon and Abu-Ghannam [31], suggested seaweed as nutraceuticals or functional foods with dietary benefits beyond their fundamental macronutrients, highlighting their significant effect on obesity and dietary related disease. This study also suggested recent developments of seaweed applications for human health from epidemiological and functional food perspectives. Brownlee et al. [32] reported the importance of alginate (algal polysaccharide) as a dietary supplement for maintenance of normal health. They also studied fiber-like activities, particularly its effects on intestinal absorption and the colon. Along with health benefits, alginate has several roles in cosmetics to help retain moisture and act as an emulsion stabilizer, bonding agent, and facial mask (filmogen) to hydrate, soothe, and soften skin. Seaweeds are similarly known as marine algae, a term encompassing macroscopic multicellular, benthic, non-flowering eukaryotic photosynthetic organisms [33,34,35,36,37]. Marine algae are found in diversified habitats such, as in tidal or sub-tidal regions, or shallow coastal water of the sea. They are also found attached with substrata, such as other aquatic plants, rocks, dead corals, pebbles, shells, and sand particles [38,39,40]. Green and red algae belong to the Plantae kingdom, whereas brown algae belong to the Chromista kingdom [41,42]. It is classified mainly in three categories based on the presence of photosynthetic pigments, such as red algae, green algae, and brown algae in Rhodophyta, Chlorophyta, and Ochrophyta phylum (Phaeophyceae class), respectively [43,44]. Many researchers suggested that marine seaweeds are effective natural alternatives to synthetic chemicals by showing many skin benefits, such as moisture retention, cell renewal activation, cell metabolism, regulation of sebaceous secretion and tissue drainage, promoting blood circulation, and increasing skin resistance [45,46,47]. There are many seaweed-based cosmetic products commercially available, such as Voya Get Glowing Illuminating Clay Mask (*Himanthalia elongata*), OSEA Ocean Cleansing Mudd (*Fucus vesiculosus*), Biossance Squalane + Probiotic Gel Moisturizer (*Chrondrus crispus*), Repêchage Vita Cura B3 Serum Complex (*Laminaria digitata*), and Ayla Sea Soak (*Macrocystis pyrifera*). Some other products, such as True Botanicals Clear Pure Radiance Oil, Skinceuticals Daily Moisture, Mario Badescu Seaweed Night Cream, and Dr. Dennis Gross Skincare Hyaluronic Marine Oil-Free Moisture Cushion, etc., also occupy the cosmetic market. In addition, Heo et al. [26] reported that seaweed is a promising and focused group of novel biochemically active principal molecules and nowadays appreciated in the developments of new biotechnological or cosmeceutical purposes.

The chemically diversified nature and unique potential of seaweeds are the reason why they have been the focus of interest for the past few years in various cosmetic applications. Seaweed-based protein, polysaccharides, phenolic compounds, and pigment profiles present cosmetic and cosmeceutical potential. This review study gives an overall view of an exploitation of seaweed for cosmetic beneficial activities. Mainly, the role of polysaccharide, protein, phenolic compounds, and pigments in different skin cosmetic beneficial activities are discussed.

### 1.1. Cosmetic Potential of the Seaweed Compounds

Marine macroalgae produce both primary metabolites, including proteins, amino acids, polysaccharides, fatty acids, etc., and secondary metabolites, such as phenolic compounds, pigments, sterols, vitamins, and other bioactive components [48,49,50,51,52,53,54]. Moreover, various types of biological activities expressed by different phycocompounds, such as blood coagulation system, antilipidemic activity, immunomodulating effect, antiviral activity, anticancer activity, antimicrobial activity, antioxidant activity, and other significant activities [55]. Especially in the area of cosmetics, many scientists reported skin beneficial activities, such as antiaging, anti-wrinkle, anti-cellulite, antioxidant, moisturizing, whitening, and photoprotection [56,57,58,59,60,61,62,63]. Sun and Chavan, [64] studied *Fucus vesiculosis* extract to reduce the appearance of dark circles on the skin area by enhancing the expression of hemeoxygenase-1. By removing heme catabolites, it eliminates the heme production on skin. Hagino and Saito [65] reported some algae species and derived compounds for UV protection benefits, skin moisturization, and inhibition of melanin synthesis. Leyton et al. [66] identified phlorotannins, pholoroeckol, and phloroglucinol in the extract of brown macroalgae *Macrocystis pyrifera*. They also reported good antidiabetic and antioxidant activity of phlorotannins, which can prevent skin aging.

Additionally, Yu and Gu [67] reported the role of algae-derived polysaccharides in the formation of protective membrane to prevent water evaporation in skin. Likewise, Sulfated polysaccharide from the red algae appear to be an excellent candidate to substitute hyaluronic acid as a bio lubricant and antioxidant [68]. Low-molecular-weight polysaccharides derived from red algae *Pyropia yezoensis* had skin beneficial functions, such as antioxidant, anti-inflammation, photoaging protection, etc. [69]. In the case of pigment, red carotenoid pigment, astaxanthin, scavenges free radicals and blocks proinflammatory cytokine production [70]. Moreover, Thomas and Kim [71] isolated fucoxanthin from *Laminaria japonica*; it is reported to inhibit tyrosinase activity and melanogenesis in UVB-irradiated mice.

### 1.2. Polysaccharides

The polysaccharides are the most significant and beneficial compounds present in macroalgae and characterized for their biological skin beneficial activity. Seaweeds are well-known for many different types of a polysaccharides, such as chitin, fucoidans, agar, carrageenan, alginates, ulvans, terpenoids, and tocopherol [72,73,74,75,76,77]. In skin cosmeceuticals, marine algae have exhibited activities such as anti-melanogenesis, antioxidant, anti-skin-aging, anti-inflammation, anti-atopic dermatitis, anti-skin-cancer, and repair of UV-induced damage [6,78,79,80,81,82,83]. A significant amount of carbohydrates is found in many macroalgal species, such as *Kappaphycus alvarezii* (formerly *Eucheuma cottonii*) (Rhodophyta), *Sargassum polycystum* (Phaeophyceae), *Padina boryana* (formerly *Padina tenuis*) (Phaeophyceae), *Fucus vesiculosus* (Phaeophyceae), *Porphyra umbilicalis* (Rhodophyta), etc. Moreover, polysaccharides have a wide variety of applications, such as photoprotection, moisturizer, wound-healing agents, thickening agents, emulsifiers, and preservatives [26,84,85,86,87,88,89,90]. Nowadays, the makers of skincare products are focused on compounds to control or regulate potential tyrosinase inhibition, inhibition of collagenase, elastase, reduction of matrix metalloproteinase (MMP) activity, reduce reactive oxygen species (ROS), and antioxidant activity [91,92,93,94,95,96]. Table 1 exhibits different potential skin beneficial effects of seaweeds’ polysaccharides. Of particular interest to the cosmeceutical utility, Fujimura et al. [28] explained the applications of purified fucoidan extracts of *Fucus vesiculosus* into creams and lotion, providing antiaging and anti-wrinkle benefits. They also reported collagenase expression, anti-inflammatory activity, and inhibition of matrix enzymes against hyaluronidase, heparinase, tyrosine kinase, and phospholipase A2. Holtkamp et al. [97] also reported the usefulness of fucoidan, particularly in skin protecting, antioxidants, antiaging, antiviral, anti-inflammatory, antitumor, and anticoagulant properties, by epidemiological and experimental studies. Fujimura et al. [98] illustrated a significant reduction in skin thickness, together with improvements in elasticity, of gel formulation with 1% Fucus extract. Additionally, Teixeira and Hellewell [99] revealed the use of fucoidan as a topical anti-inflammatory for cosmetic after-sun damage, allergic-condition-soothing products, or specially postsurgical formulations. Polysaccharides have also been widely shown to exhibit antioxidant, antiviral, anticoagulant, and antitumor properties in commercial products. For instance, lipid soluble fraction of an edible red alga *Gelidium amansii* to induced apoptosis of cancer cells in vitro [100]. Like fucoidan, carrageenan produces a range of textures for creams, lotions, sticks, sprays, and foams formulation [101]. It has a range of biological properties and is applicable in various pharmaceutical properties, such as antitumor, immunomodulation, anti-hyperlipidemic, and anticoagulant activities [102]. Another phenolic compound are alginates found in the cell wall of brown algae (Phaeophyceae), mainly *Laminaria* species (*Laminaria hyperboreun*, *Laminaria digitata*, *Laminaria japonica*) and also *Macrocystis pyrifera*, *Ascophyllum nododsum*, *Ecklonia maxima*, *Lessonia nigrescens*, *Ascophyllum nodosum*, *Durvillea antarctica*, and *Sargassum* sp. [98,103]. Podkorytova et al. [104] also employed alginates in cosmetics for face-mask and body-wash ingredients due to their benefits to the skin structure and function. Specifically, alginates are highly efficient when used to solidify and stabilize emulsion at a low pH [101]. Skjak-Bræk et al. [105] suggested inhomogeneity of low-molecular-weight alginate in gel formulation with low concentration of Ca^+2^ and absence of non-gelling ions Na^+^.

There are a wide variety of polysaccharides that are useful in skin cosmetics, such as agar, alginic acid, carrageenan, porphyrin, laminarin, fucoidan, and ulvan. Many genera of agrophytes algae, such as *Gelidium* sp., *Gracilaria* sp., *Gelidiela* sp., *Pterocladiella* sp., etc., are well-known producers of agar-agar [51,134]. Balboa et al. [135] suggested use of agar as a major ingredient in creams, as an emulsifier, stabilizer, moisturizer as well as in different cosmetic products such as lotion, deodorants, antiaging treatment, exfoliant, acne treatment, etc. Like agar, alginic acid is derived from several brown algal species (Fucales, Laminariales, *Ascophyllum* sp., *Durvillaea* sp., *Ecklonia* sp., *Laminaria* sp., *Macrocystis* sp., *Saccharina* sp., *Sargassum* sp., and *Turbinaria* sp.) [136,137,138]. Mafinowska [139] and Fabrowska et al. [140] reported its application in the formulation of skin-protective or barrier creams for the treatment of dermatitis, as well as suitable ingredients of beauty masks or facial packs. In addition, Kappa-, Iota-, Lambda-, Beta-carrageenan are extracted from several carrageenophytes, i.e., *Betaphycus gelatinum*, *Chondrus crispus*, *Eucheuma denticulatum*, *Gigartina* sp., *Kappaphycus alvarezii*, *Hypnea musciformis*, *Mastocarpus* sp., and *Mazzaella* sp., from the Rhodophyta. It is used in cosmetology for various applications, such as lotion, sun-ray protectors, medicines, deodorant sticks, sprays, and foams [141,142,143,144].

Moreover, porphyrin is a well-studied class of sulfated polysaccharides obtained from the aqueous extract of red algae *Porphyra* sp. and *Bangia* sp. [145,146]. It has shown potential cosmeceutical applications, such as skin-whitening, antiulcer, analgesic, and anti-inflammatory properties. Many brown seaweed species, including *Laminaria* sp., *Saccharina* sp., *Ascophyllum* sp., *Fucus* sp., *Sargassum* sp., and *Undaria* sp., are well-known for laminaran properties, such as antitumor, anti-inflammatory, antiviral, antioxidant, anticoagulant, and anti-cellulite properties [147,148,149]. Among all, sulfated polysaccharides have attractive considerable attention in cosmeceutical activities: UV protector, anti-inflammatory, anticoagulant antithrombotic, tyrosinase inhibitor, antitumoral, antibacterial, antidiabetic, and antioxidative [150,151,152,153]. Moon et al. [154] found the role of fucoidan in an inhibition of matrix metalloproteinase induced by UVB radiation. Accordingly, Senni et al. [155] suggested its potential role in prevention of photoaging of the skin. Consequently, fucoidan acts as an inhibitor of tyrosinase and reduces skin pigmentation, while used in skin-whitening formulation [156,157]. Pereira [158], Carvalho and Pereira [159], and Gesztesi et al. [160] suggested ulvan as desirable raw material for cosmeceuticals. Apart from, ulvans have beneficial moisturizing, protective, antitumor, and antioxidative properties in gel formulation [161,162]. Yaich et al. [163] described the skin protective and bioactive effects of rhamnose and fucose against skin aging. The unique chemical and physiochemical properties of polysaccharides make them attractive candidates for novel functional and biologically active polymer for cosmeceuticals [134].

### 1.3. Proteins

Biological macromolecule protein is a polymer of amino acids that is present in all living organisms. It is a basic building block of almost all cellular processes. It may present itself in the form of enzymes, hormones, vitamins, and pigments [164,165]. Moreover, macroalgae contain different types of aliphatic amino acids, hydroxyl-group-containing amino acid, aromatic amino acid, mycosporine amino acids, etc., which are summarized in Table 2. In addition, different species of *Palmaria palmata*, *Chondrus crispus*, *Porphyra* sp. (Rhodophyta), *Undaria pinnatifida* (Phaeophyceae), *Ulva* sp. (Chlorophyta), and *Euchema* sp. (Rhodophyta) are reported for the quantity of amino acids they contain [166,167,168]. It is widely applicable in cosmeceutical preparation as a functional part. It exhibits many cosmeceutical activities, such as anti-inflammatory, antiaging, antioxidant, and photoprotection activities [169,170]. According to Fabrowska et al. [171], protein showed a moisturizing effect on human skin. MAAs (mycosporine-like amino acids) play their role in the absorption of solar energy that beneficiary in photoaging, as well as photo-damaging protection. There are different roles of MAAs, such as in UV protection, anti-photoaging, antioxidant, and anti-hypertensive activities, reported by researchers [172,173,174,175]. Moreover, Dunalp and Yamamoto [176] reported the importance of mycosporine amino acids (MAAs) as sunscreens to reduce UV-induced damage. MAAs play a major role in protection against damage caused by sunlight. They acting as antioxidant molecules which scavenge toxic oxygen radicals and protect skin against UV-induced damage [177]. Furthermore, MAAs act as protective solutes of cells against salt stress, desiccation, and thermal stress [178].

Galland-Irmouli et al. [196] and Samarakoon and Jeon [197] reported various skin-benefiting activities of protein and amino acids: anti-inflammatory, antioxidant, antitumor, antiaging, skin protective, and moisturizing effects in cosmetic products and the natural moisturizing factor in human skin. Houston [198] found *Ulva australis* to be a good source of essential amino acids, such as histidine and taurine. whereas Galland-Irmouli et al. [199], Pereira [200], and Martínez–Hernández et al. [201] have shown that red alga *Palmaria palmata* and *Himanthalia elongata* are a high source of serine, alanine, and glutamic acid. Reef et al. [202] and Pereira, [203] detected mycosporines, such as amino acids (MAAs), in different red macroalgae (Rhodophyta): *Chondrus crispus*, *Palmaria palmata*, *Gelidium* sp., *Porphyra* sp., *Gracillaria cornea*, *Asparagopsis armata*, *Grateloupia lanceola*, and *Curdiea* sp. Pereira, [203] showed role of MAAs as UV protectors and activators of cell proliferation in cosmetics.

The red alga *Porphyra rosengurtii*–derived mycosporine-like amino acids Porphyra-334 and Shinorine are isolated and found to be very photostable and photoprotective when exposed to radiation [204]. These MAAs both played a role in sunburn cell formation and to be protective after UV radiation and eliminate damaged cells [205]. This combination also used in treatment of prevention towards skinfold thickening in the epidermis/dermis of hypodermic of mice.

### 1.4. Phenolic Compounds

Marine macroalgae are richer in various phenolic compounds, such as catechins, flavonols, flavonolglycosides, phloroglucinol, gallic acid, epicatechin, pyrocatechol, gallate, flavonoids, anthocyanins, stilbenes, lignans, and phenolic polymers [206,207]. These types of phenolic compounds revealed their effect on MMP (Matrix Metalloproteinase complex) inhibition, as well as the reduction of collagen degradation [208]. This research also reported algal-derived phenolic compounds helpful to suppress both the protein and gene expression of the MMP complex. Ryu et al. [209] suggested that *Corallina pilulifera* (Rhodophyta) can inhibit the expression of MMP-2 and MMP-9. Another phenolic compound, sargachromanol E, from *Sargassum horneri* (Phaeophyceae), expressed its effect on antiaging activity [210]. Porphyra 334, a mycosporine amino acid from *Phycocalidia vietnamensis* (formerly *Porphyra vietnamensis*) (Rhodophyta), showed UV-absorbing properties [211]. Catechin and some other phycocompounds, such as flavonoids, polyphenol, and carotenoids, showed ROS scavenging, downregulation of the mitogen-activated protein kinase (MAPK) pathway, inhibition of MMP, and the elevation of collagen production, giving them wider application in cosmetics [212,213]. Moreover, brown macroalgae *Ecklonia cava*-derived compounds, such as phlorotannins, exhibit skin whitening/antityrosinase effect, whereas zeaxanthin from the microalga *Nannochloropsis oculata* (Ochrophyta and Eustigmatophyceae) extracts showed skin-whitening activity [214]. The beneficial activities of seaweed-derived phenolic compounds for skin uses are illustrated in Table 3. Pavia and Brock [215] and Bravo [216] identified phenolic compounds such as phlorotannins and phloroglucinol (1,3,5-trihydroxybenzene) in different brown algal families, such as Alariaceae, Sargassaceae, and Fucaceae. Different algal species are evaluated for antioxidant activity by different methods, such as DPPH (2,2-diphenyl-1-picrylhydrazyl) radical scavenging activity, ferrous ion-chelating ability, and ORAC (Oxygen Radical Absorbance Capacity) [217,218]. Ferreres et al. [219] and Sanjeewa et al. [220] reported anti-allergic, anti-wrinkle, and skin antiaging activities of phlorotannins, due to hyaluronidase inhibition activity. Jang et al. [221] studied tyrosinase inhibition and the whitening effect of phlorotannins from *Sargassum fusiforme* (*Hijikia fusiformis*). Phlorotannins, eckol, Fucols, Fucophorethols, Fuhalols, Phlorethols from *Corallina pilulifera* (red algae) have beneficial cosmetic properties: antiaging, antiphotoaging, antioxidant, anti-allergic, anti-inflammatory, tyrosinase inhibition, and hyaluronidase inhibition [222,223,224,225]. Moreover, phlorotannins are reported to be inhibitors of melanin synthesis, as well as being protective against UVB photodamage [226]. Likewise, Handelman [227] and Wang et al. [228] revealed the inhibitory effect *Ecklonia cava*–derived phlorotannins on melanin synthesis and protective effects on UV damage.

### 1.5. Pigments

Macroalgae is cultivated in a controlled condition to regulate the production of bioactive compounds such as phenolic compounds, pigments, carbohydrates, proteins, amino acids, vitamins, and minerals [259]. These algae-based valuable bioactive constituents gained attention in cosmeceutical activities [260]. This algae-derived metabolite can repair early signs of skin-aging, has an anti-wrinkle effect, exerts tightening effects, collagen synthesis, etc., as reported from *Arthrospira* species (Cyanobacteria) and *Chlorella valgaris* (Chlorophyta) [261,262]. Marine algae contain a broad range of photosynthetic pigments chlorophylls, carotenoids (carotenes, xanthophylls, fucoxanthin, and peridinin), and phycobilins (phycocyanin and phycoerythrin) [263,264]. As suggested by many researchers, red algae contain chlorophyll, phycobilin, carotenoids, β carotene, lutein, phycocyanin, and phycoerythrin Whereas brown algae possess chlorophyll a, c, carotenoids, fucoxanthin, and other pigments. Likewise, Chlorophyta revealed the presence of chlorophyll-a, -b, and -c and carotenoids [265,266,267,268,269,270,271]. Due to the richness of diversified pigments’ profile, it is applied in various applications, such as photoprotection, anti-inflammatory effects, anticancer effects, and the inhibition of cell proliferation [272,273,274,275,276]. The benefits of seaweed-derived pigments are summarized in Table 4. According to Takaichi S. [277], Quilodrán et al. [278], and Amon and French [279], algae species are considered as a major source of β-carotene; likewise, some compounds, such as carotenoids, astaxanthin, and docosahexaenoic acid (DHA), show antioxidant activity. Hosikian et al. [280] evaluated the role of green photosynthetic pigments in cosmetic industrial applications for antioxidant and antimutagenic properties. Spears [281] and La-Mer [282] suggested role of chlorophyll as natural coloring agents, deodorizing and antibacterial properties. In addition, these chlorophylls have high antioxidant activity and the ability for tissue-growth stimulation, making them useful to the cosmetic industry [283,284].

Carotenoids are widely applicable as natural dyes and antioxidants with antitumor, anti-inflammatory, and radical sequestering benefits [285,286,287]. They modulate UVA-induced gene expression and protect the skin against UV light [288]. Moreover, astaxanthin has a variety of roles in the prevention of UV-mediated photo-oxidation, tumors, and inflammation [289]. Additionally, fucoxanthin has protective effects on skin, making it consequently beneficial in cosmetics [290,291] Likewise, Kushwaha et al. [292] and Morabito et al. [282] reported carotenoids having antioxidant and anti-inflammatory properties that help for photoprotection and against UVA-damaging effects.

**Table 4:** Skin beneficial activities of marine macroalgae’s pigments.

No.	Species	Potential Pigment/s Studied	Cosmetics Properties and/or Products	References
1	*Chaetomorpha antennina*, *Padina gymnospora*	Chlorophyll, Carotenoid, Xanthophylls, Antioxidant	Photoprotection	[283]
2	*Sargassum aquifolium* (formerly *Sargassum binderi*),	Fucoidan	Photoprotection	[284,285]
3	*Ulva lactuca,* *Caulerpa racemosa*, *Bryopsis plumosa*, *Gelidiella acerosa*, *Hypnea valentiae*	Chlorophyll Carotenoid	Photoprotection	[286]
4	*Sargassum ilicifolium*	Fucoxanthin	Photoprotection Antioxidant	[287]
5	*Sargassum polycistum*	Fucoxanthin β carotene α carotene	Antioxidant	[288]
6	*Haematococcus lacustris* (formerly *Haematococcus pluvialis*)	Lutein β carotene	Photo-oxidative	[289]
7	*Sacharina latissima* (formerly *Laminaria saccharina*)	Chlorophyll	Photo-inhibition	[290]
8	*Chondrus crispus*	Carotenoid	Photoprotection	[291]
9	*Kappaphycus alvarezii*, *Padina australis*	Chlorophyll a β carotene Fucoxanthin Zeaxanthin	Photoprotection	[292]

## 2. Discussion

Cosmetic researchers have focused their attention on marine organisms as an additional source of novel and useful natural ingredients. Diversified marine-algae-derived secondary metabolites are structurally more complex, with unique functionalities and properties. This review surveyed the potential applications of marine-algae-derived compounds for various skin benefits in the cosmetic industry. Though many seaweeds are exploited for their cosmetic properties, the research work on them is still incomplete, and so many species, either in full or in part, have not been explored. Hence, the cost-effective and efficient alternative standardized method to extract the bioactive phyco-constituents with significant productivity and activity is in growing demand. In future perspectives, the responsible molecular mechanism and safety concerns of these compounds are very important for future challenges in cosmeceuticals. Therefore, further investigations to study the precise molecular basis for the beneficial activity of marine algal components should be undertaken. Recently, in silico tools and techniques have been used to select functional materials derived from natural resources quickly and to predict the mechanisms of actions. Thus, this approach will be a helpful strategy for finding and understanding more effective compounds with the novel property.

## 3. Conclusions

The overexposure of human skin to different environmental stresses, such as pollutants and sun radiation, as well as chemical cosmeceutical ingredients—it increases the production of reactive oxygen species (ROS)—leads to many skin-damaging problems, such as aging, dullness, carcinogenesis, wrinkles, age spots, dark circles, etc. Marine-algae-based bioactive purified compounds demonstrated highly significant beneficiary applications in cosmetic formulas, as multiple functions, where they can be natural active constituents to the synthetic ingredients. Under different environmental factors, marine algae have the biosynthesis of primary and secondary metabolites for their survival. These biologically active constituents can be used as an active ingredient in the cosmetic industries due to their various skin benefits. It could be used as an antioxidant, antimicrobials, antibacterial, whitening agent, antiaging, anti-wrinkle, anti-acne, moisturizing, UV protection, deodorizing, anti-allergic, anti-inflammatory, sensory enhancer, viscosifying, stabilizer, and also for thickening in cosmetic industries. Sustainable use of marine algae and marine-algae-based molecules is crucial for humankind. Moreover, there are many cosmeceutical industries that already use extracts of marine algae and compounds in the formulation of many products. However, the monitoring of its biochemical profile presents a problem that needs to overcome. This can be solved by the development of seaweed cultivation and green extraction methods that are being analyzed with promising research results. However, many cosmetic companies’ collaboration at the national and international level can improve the analytical methods of its screening for safety, thus enhancing consumer’s safety towards marine-algae-based bioactive compounds in the cosmetic products. All mentioned marine algae in this review, possessing various bioactivities, are considered and utilized as a natural inexhaustible source for different cosmeceutical benefits.

## Figures and Tables

**Table 1:** Skin-benefiting activities of polysaccharides derived from marine macroalgae.

No.	Species	Cosmetics Properties and/or Products	References
1.	*Ecklonia cava*	Anticoagulant activity	[106]
2.	*Ishige okamurae*, *Schizymenia dubyi*, *Ecklonia cava*, *Ecklonia stolonifera*, *Sargassum silauastrum*	Tyrosinase inhibition	[107,108,109]
3.	*Sargassum fusiforme* (formerly *Hizikia fusiformis*)	Collagenase and elastase inhibition	[110]
4.	*Saccharina japonica* (formery *Laminaria japonica*)	Antioxidant activity	[111]
5.	*Neoporphyra haitanensis* (formerly *Porphyra haitanensis*), *Ulva australis* (formerly *Ulva pertusa*), *Ulva linza* (formerly *Enteromorpha linza*), *Bryopsis plumosa*	Antioxidant activity	[112]
6.	*Sargassum* sp., *Neopyropia yezoensis* (formerly *Porphyra yezoensis*)	Antilipidemic activity	[113,114]
7.	*Fucus* sp., *Laminaria* sp., *Sargassum* sp.	Skin-whitening effect	[115,116,117]
8.	*Turbinaria ornata*	Antioxidant, anti-inflammatory	[118]
9.	*Sargassum polycystum*	Tyrosinase inhibition	[115]
10.	*Corallina pilulifera*, *Ecklonia cava*	Inhibition of MMP-2,9	[81,119]
11.	*Schizymenia binderi*	Antiviral activity	[120]
12.	*Fucus vesiculosus*, *Turbinaria conoides*	Antioxidant (photoprotection)	[121]
13.	*Gongolaria nodicaulis* (formerly *Cystoseira nodicaulis*), *Eisenia bicyclis*, *Ecklonia cava* subsp. *kurome* (formerly *Ecklonia kurome*)	Hyaluronidase inhibition Antiaging	[122,123]
14.	*Undaria pinnatifida*, *Codium tomentosum*, *Durvillaea antarctica*, *Saccharina japonica* (formery *Laminaria japonica*), *Ulva* sp.	Moisture retention	[120,124,125]
15.	*Sargassum patens*	Antiviral activity	[126]
16.	*Lobophora variegata*	Antiviral activity	[127]
17.	*Sargassum vulgare*, *Colpomenia sinuosa*, *Dictyopteris polypodioides* (formerly *Dictyopteris membranacea*)	Antimicrobial	[128]
18.	*Padina pavonica*, *Ecklonia cava*	Antimicrobial activity	[129]
19.	*Corallina* sp., *Saccharina japonica* (formerly *Laminaria japonica*)	Antimicrobial Moisturizer	[130,131]
20.	*Porphyra umbilicalis*	Reduced ROS by UV	[132,133]

**Table 2:** Skin-benefiting activities of proteins/amino acids derived from marine macroalgae.

No.	Species	Cosmetics Properties and/or Products	References
1.	*Laminaria digitata*	Lipolytic activity	[179]
2.	*Porphyra umbilicalis*	Anti-UVA Antioxidant	[180] [181]
3.	*Ecklonia cava*	Antioxidant, chelating agent, radical scavenger	[182]
4.	*Palmaria palmata*, *Neopyropia yezoensis* (formerly *Porphyra yezoensis*), *Ulva prolifera* (formerly *Enteromorpha prolifera*)	Antioxidant activity	[183]
5.	*Sargassum polycystum*	Anti-melanogenesis/skin-whitening effect	[184]
6.	*Pelvetia canaliculata*	Antioxidant, collagen synthesis,	[185]
7.	*Jania rubens*	Anti-skin-cancer	[186]
8.	*Porphyra umbilicalis*	Sunscreen	[187]
9.	*Scytosiphon lomentaria*	Antioxidant	[188]
10.	*Furcellaria lumbricalis*, *Fucus vesiculosus*	Anti-skin-aging	[189]
11.	*Acanthophora nayadiformis*	Antioxidant, radical scavengers	[190]
12.	*Limnospira maxima* (formerly *Spirulina maxima*), *Ulva lactuca*, *Rhizoclonium riparium* var. *implexum* (formerly *Lola implexa*)	Anti-skin-aging	[191]
13.	*Palmaria palmate*	Moisturizer, natural sunscreen, antioxidant	[192]
14.	*Neopyropia elongata* (formerly *Porphyra rosengurttii*)	Photoprotective effects	[193]
15.	*Ecklonia stolonifera*	Inhibition of MMP	[194,195]

**Table 3:** Skin beneficial activities of phenolic compounds derived from marine macroalgae.

No.	Species	Potential Phenolic Compound/s Studied	Cosmetics Properties and/or Products	References
1	*Sargassum muticum*, *Ishige okamurae*, *Ecklonia cava, Polysiphonia morrowii*, *Dictyopteris undulata*, *Sargassum micracanthum*, *Sargassum macrocarpum*	Total phenolic compounds	Antioxidant	[229]
2	*Acetabularia ryukyuensis*, *Undaria pinnatifida*, *Gelidium elegans*	Flavonoid	Antioxidant	[230]
3	*Sargassum siliquastrum*	Total phenolic compounds	Antioxidant	[231]
4	*Ascophyllum nodosum*	Phlorotannin Flavonoid	Antioxidant	[232,233]
5	*Ulva prolifera* (formerly *Enteromorpha prolifera*)	Flavonoid	Anti-inflammatory, Antiviral, Anticoagulant	[234]
6	*Fucus serratus*, *Sargassum muticum*, *Saccharina latissima*, *Laminaria digitata*, *Agarophyton vermiculophyllum* (formerly *Gracilaria vermiculophylla*)	Phenolic compound	Antioxidant	[235]
7	*Anthophycus longifolius*, *Sargassum plagiophyllum*, *Sargassum myriocystum*	Total Phenolic compounds	Antioxidant	[236]
8	*Chaetomorpha antennina*	Phenolic compound	Antiviral, Antibacterial, Antifungal, Anticancer	[237]
9	*Fucus vesiculosus*	Polyphenol	Antioxidant	[238]
10	*Halimeda macroloba*, *Halimeda opuntia*	Phenolic acid (compound)	Antioxidant	[239]
11	*Gongolaria barbata* (formerly *Cystoseira barbata*), *Scytosiphon lomentaria*, *Chondracanthus acicularis* (formerly *Gigartina acicularis*)	Phenolic compounds Carotenoids Flavonoids	Antioxidant	[240]
12	*Halimeda monile*	Phenolic acid	Antioxidative	[241]
13	*Fucus vesiculosus*, *Ascophyllum nodosum*, *Fucus serratus*	Polyphenols Phlorotannins	Antioxidant	[242]
14	*Sirophysalis trinodis* (formerly *Sirophysalis trinodis*)	Phlorotannins	Antioxidant	[243]
15	*Ecklonia cava*	Phloroglucinol	Antioxidant	[244]
16	*Fucus vesiculosus*, *Ascophyllum nodosum*, *Fucus serratus*,	Phlorotannins	Antioxidant	[245]
17	*Ceramium rubrum*, *Cladophora vagabunda*, *Ulva intestinalis* (formerly *Enteromorpha intestinalis*)	Phenolic compound	Antioxidant	[246]
18	*Ulva lactuca*	Phlorotannins	Antioxidant	[247]
19	*Sargassum fusiforme* (formerly *Hizikia fusiformis*), *Ishige foliacea*	Phlorotannins	Tyrosinase inhibition, Antioxidant, Anti-Inflammatory, Anti-allergic	[248,249]
20	*Ecklonia stolonifera*, *Eisenia bicyclis*	Phlorotannins	Anti-inflammatory, Antioxidative	[250]
21	*Vertebrata thuyoides* (formerly *Boergeseniella thuyoides*), *Gracilaria multipartita*	Phenolic compound and Flavonoids	Antioxidant	[251]
22	*Sargassum pacificum* (formerly *Sargassum mangarevense*), *Turbinaria ornata*	Phenolic compound	Antioxidant, Antimicrobial	[252]
23	*Cladophora rupestris*, *Codium fragile*	Phenolic compound	Antioxidant, Mineralogenic	[253]
24	*Sargassum siliqastrum*	Fucoxanthin	Antioxidant	[254]
25	*Desmarestia ligulata*, *Dictyota kunthii*, *Laurencia chilensis*, *Chondracanthus chamissoi*	Flavonoids Polyphenols	Antioxidant, Cytotoxic, Anticancer	[255]
26	*Sargassum pacificum* (formerly *Sargassum mangarevense*), *Turbinaria ornata*	Phenolic compound	Antioxidant, Antimicrobial	[254]
27	*Ericaria selaginoides* (*Cystoseira tamariscifolia*)	Phenolic compound	Photoprotection	[256]
28	*Eisenia arborea*	Phlorotannins	Anti-inflammatory	[257]
29	*Pyropia columbina* (formerly *Porphyra columbina*)	Phenolic compound	Antioxidant	[258]

## Data Availability

Not available.
